# A Spectrum of Pathogenic Variants in the *LAMA2* Gene in the Russian Federation

**DOI:** 10.3390/ijms26031257

**Published:** 2025-01-31

**Authors:** Polina Chausova, Tatiana Cherevatova, Elena Dadali, Aysylu Murtazina, Maria Bulakh, Sergei Kurbatov, Inga Anisimova, Ilya Kanivets, Vasilisa Udalova, Galina Rudenskaya, Nina Demina, Inna Sharkova, Anastasia Monakhova, Polina Tsygankova, Tatiana Markova, Oksana Ryzhkova, Olga Shatohina, Varvara Galkina, Artem Borovikov, Irina Mishina, Olga Shchagina, Alena Chukhrova, Aleksander Polyakov

**Affiliations:** 1 Research Centre for Medical Genetics, Moscow 115522, Russia; tatiana_milovidova@mail.ru (T.C.); genclinic@yandex.ru (E.D.); aysylumurtazina@gmail.com (A.M.); mariya.bulakh@gmail.com (M.B.); anisimova-inga@med-gen.ru (I.A.); rudenskaya@med-gen.ru (G.R.); ndemina47@mail.ru (N.D.); sharkova.inna@gmail.com (I.S.); polgamma@yandex.ru (P.T.); markova@med-gen.ru (T.M.); ryzhkova@dnalab.ru (O.R.); mironovich_333@mail.ru (O.S.); vgalka06@rambler.ru (V.G.); borovikov33@gmail.com (A.B.); akimova@med-gen.ru (I.M.); schagina@dnalab.ru (O.S.); achukhrova@yandex.ru (A.C.); apol@dnalab.ru (A.P.); 2 Research Institute of Experimental Biology and Medicine, Voronezh State Medical University N.N. Burdenko, Voronezh 394036, Russia; kurbatov80@list.ru; 3 Department of Neurology named after K.N. Tretyakov, Saratov State Medical University, Saratov 410000, Russia; 4 Genomed LLC, Moscow 105005, Russia; dr.kanivets@genomed.ru (I.K.); udalova@genomed.ru (V.U.); 5 Veltischev Research and Clinical Institute for Pediatrics and Pediatric Surgery, Pirogov Russian National Research Medical University, Moscow 125412, Russia; stasya1803@mail.ru

**Keywords:** *LAMA2*-associated muscular dystrophy, congenital muscular dystrophy, *LAMA2* gene, CMD

## Abstract

*LAMA2*-associated muscular dystrophy is a rare genetic disorder caused by pathogenic or likely pathogenic variants in the *LAMA2* gene. The aim of this study is to characterize the spectrum of pathogenic/likely pathogenic variants in the *LAMA2* gene among Russian patients, identify frequent pathogenic variants specific to this population, and estimate the prevalence of this disorder in Russia. Data were collected and analyzed from patients with confirmed diagnoses of *LAMA2*-associated muscular dystrophy using various molecular genetic methods in research centers from 2008 to 2024. Data were obtained from 90 unrelated patients with *LAMA2*-associated muscular dystrophy, out of which 83 presented with the more severe form, MDC1A1, while seven had milder form of *LAMA2*-associated muscular dystrophy. The most common pathogenic variants identified were nonsense mutations (40% of cases), followed by frameshift variants (29.3%), splicing variants (21.4%), gross deletions (5.3%), and missense variants (4%). It is worth noting that missense variants were found exclusively in patients with the milder form of *LAMA2*-associated muscular dystrophy. The most prevalent identified pathogenic variant was c.7536del (15%), characteristic of Slavic populations with an established founder effect. Additionally, a common pathogenic variant, c.8245-2A>G, was found predominantly in Kazan Tatars. The estimated prevalence of *LAMA2*-associated muscular dystrophy in Russia is approximately 1 in 117,700.

## 1. Introduction

*LAMA2*-associated muscular dystrophy is a rare neuromuscular disorder with an autosomal recessive inheritance pattern caused by pathogenic or likely pathogenic variants in the *LAMA2* gene. This gene, mapped to 6q22.33, consists of 65 exons and encodes the α2-subunit of the extracellular heterotrimeric protein laminin-211 (also known as laminin-2 or merosin). The absence of this protein or its functionally inactive form leads to the development of muscular dystrophy [1]. Clinically, two forms are distinguished: a more severe form, congenital merosin-deficient muscular dystrophy (muscular dystrophy, congenital, merosin-deficient or partially deficient, MDC1A, OMIM_607855), and a milder form, limb-girdle muscular dystrophy (muscular dystrophy, limb-girdle, autosomal recessive 23, LGMDR23, OMIM_618138). The prevalence of the more severe form is estimated to be 0.6–0.7 per 100,000 in the United Kingdom and Italy [2]. The prevalence of the milder form has not been estimated. However, studies conducted in the United Kingdom and Denmark indicate that pathogenic or likely pathogenic variants in *LAMA2* were identified in 2–3% of patients with limb-girdle muscular dystrophy [2].

This phenotypic diversity is primarily explained by the type and location of mutations within the gene, as well as by the residual merosin protein levels in affected muscles [3,4,5]. However, some authors also suggest the presence of other factors influencing the severity and progression of the disease [5,6]. The most common pathogenic or likely pathogenic variants in the nucleotide sequence of *LAMA2* are loss-of-function (LoF) variants. In cases associated with LoF variants in a homozygous or compound heterozygous state, immunohistochemical analysis of skeletal muscle biopsy reveals an absence of the laminin α2 protein [3,7]. Missense variants, in contrast, are associated with the milder form of *LAMA2*-associated myopathy. However, if missense variants are located in the G-domain, which interacts with integrins and α-dystroglycan, they can also lead to the development of MDC1A [7,8].

Currently, there are no effective treatment methods for *LAMA2*-associated muscular dystrophy, although active research is underway to develop gene therapy. Therefore, it is important to understand the spectrum of pathogenic variants in the *LAMA2* gene specific to different countries, as well as the disease prevalence. This study presents the spectrum of pathogenic or likely pathogenic variants in patients with *LAMA2*-associated muscular dystrophy residing in the Russian Federation and provides an assessment of its prevalence within the region.

## 2. Results

### 2.1. Clinical Data

At the Research Centre for Medical Genetics (RCMG) and LLC “Genomed”, 90 unrelated patients (Supplementary Table S1) with *LAMA2*-associated muscular dystrophy were identified. A total of 43 (47.7%) of them were female, and 47 (52.3%) were male.

In 83 cases, a more severe form—MDC1A—was diagnosed. All patients with MDC1A exhibited pronounced diffuse muscle hypotonia, motor development delay, joint contractures, and most of the patients were unable to walk independently. In 73/79 patients, the disease manifested at birth, in 4—at the age of one month, and in 2—at the age of three and four months, which is consistent with literature data regarding the onset period of MDC1A [2,9]. Apgar scores at birth were available for 28 out of 83 cases, with the most common score being 7/8. Birth complications occurred or cesarean sections were carried out in 9/31 cases, and 12/30 cases required a transfer to the neonatal pathology department. Creatine kinase (CK) levels were known for 48 out of 83 patients, ranging from 287 U/L to 6000 U/L (normal range is 24 to 195 U/L), though one patient had CK levels of 48 U/L [10]. In 25/29 cases, brain magnetic resonance imaging (MRI) showed signs of periventricular leukopathy; in one case, frontotemporal cortical atrophy was observed, which can also be seen in *LAMA2*-associated MD patients [2]; in one case, a congenital CNS anomaly, Dandy–Walker malformation, was detected along with leukomalacia in the cerebral hemispheres and asymmetric occlusive hydrocephalus; in one case, a congenital brain malformation was observed; and in one case, ventriculomegaly was observed. In 11/11 cases where skeletal muscle biopsy was conducted, dystrophic changes were noted. In 5/11 cases with immunohistochemistry, merosin deficiency was identified. In 17/26 cases with electromyography (EMG), a myogenic pattern was observed; in three cases, neurogenic pattern was detected; in six cases, EMG study showed no changes.

A milder clinical phenotype was diagnosed in 7 out of 90 cases.

In the first case (Patient 84), the proband was a 17-year-old boy born at full term. His birth weight was 3380 g and length was 52 cm with an Apgar score of 7/7. He was transferred to the neonatal pathology department and discharged on day 15. Motor development was delayed; he did not sit on his own and began walking at 2.5 years, with a waddling gait and frequent falls. He never ran or stood up from a squatting position without support. He had weakness in proximal muscle groups was progressing, especially during growth spurts. At the age of 13, spinal rigidity and deformity, as well as large joint contractures, were detected. Additionally, ophthalmoparesis, thigh and posterior calf muscle atrophy were noted. CK level at 8.5 years was 1437 U/L. An EMG indicated a predominantly myopathic pattern, and brain MRI showed leukodystrophy.

In the second case (Patient 85), the proband, a girl aged 3 years and 4 months, was born at full-term following a delivery complicated by shoulder dystocia and vacuum extraction. Her birth weight was 4300 g and length was 54 cm with an Apgar score of 3/5. She stayed in the neonatal pathology department and was discharged on day 15. Her motor development was delayed: sitting at nine months, walking with support at two years, and walking independently at 2.5 years. From birth, hypotonia was present. Calf muscle tightness, Gowers sign, pes valgus, along with winged scapulae were noted. She could not jump or run. Her CK levels ranged from 1600 to 2800 U/L. An MRI revealed white matter involvement in the cerebral hemispheres and spinal cord, suggesting a neurodegenerative disorder, possibly metachromatic leukodystrophy.

In the third case (Patient 86), the proband was an 11-year-old boy born at full-term. His birth weight was 2940 g and length was 52 cm. During the first year of life, motor development was mildly delayed, he began walking at 16 months. He was persistently hypotonic and rarely ran. He had an asthenic build, unaltered gait, mild lordosis, inability to walk on heels, mild calf muscle hypertrophy, and slight hypotrophy in the limb-girdle muscles. His tendon reflexes were reduced, while muscle strength was preserved. No myopathic signs were present. His CK level was 1872 U/L. A brain MRI suggested a demyelinating process or post-hypoxic changes with gliosis.

In the fourth case (Patient 87), the proband was a 40-year-old woman born at full-term with a birth weight of 2600 g and length of 52 cm. In the nursing home, her condition was normal, she was discharged at term. Her development was normal until the age of one year; she began walking at 15 months with a clumsy, waddling gait but could run and climb stairs. From age 17, muscle weakness in her legs and difficulty rising from a squat appeared. At ages 26 and 37, she gave birth to healthy daughters. After age 37, she had difficulty climbing stairs and needed support to go outside. An EMG showed a myopathic pattern, and an MRI revealed diffuse symmetrical white matter MR signal elevation in the frontal, parietal, and occipital lobes.

In the fifth case (Patient 88), the proband was a 5-year-old boy, born at full-term with a birth weight of 3280 g, a length of 50 cm, and an Apgar score of 8/9. Hypotonia was noted at the age of one month, motor development was delayed; he began walking at 16 months with a waddling gait and frequent falls. His CK level was 1515 U/L, and an EMG showed primary muscle involvement with signs of axonal-demyelinating lesions. A brain MRI showed focal white matter changes.

In the sixth case (Patient 89), the proband was a 15-year-old girl with delayed motor development from birth; she began walking at 2.5 years with a waddling gait. At the age of 15 years, she could walk independently but could not rise from a sitting position. Notable findings included muscle hypotrophy, diffuse hypotonia, large joint contractures, pronounced lumbar hyperlordosis, Gower’s sign, and waddling gait, with intact facial muscles. Her CK level at age 13 was 780 U/L. A brain MRI indicated a demyelinating brain disease (leukodystrophy), and immunohistochemical analysis (IHCA) showed a negative reaction to laminin.

In the seventh case (patient 90), the proband was a 26-year-old woman, born at full-term with a birth weight of 3400 g. She had mild motor development delay, hypotonia, a waddling gait, and toe walking. At the time of examination, she could not rise from a sitting position or climb stairs. She had Trendelenburg gait with hyperlordosis. Her CK level was 498 U/L, and an EMG showed a myopathic pattern.

### 2.2. Genetic Analysis

All 90 patients had pathogenic/likely pathogenic biallelic variants in the *LAMA2* gene, which were identified through various molecular genetic methods (see Supplementary Table S1 and Table 1).

**Figure 1:**
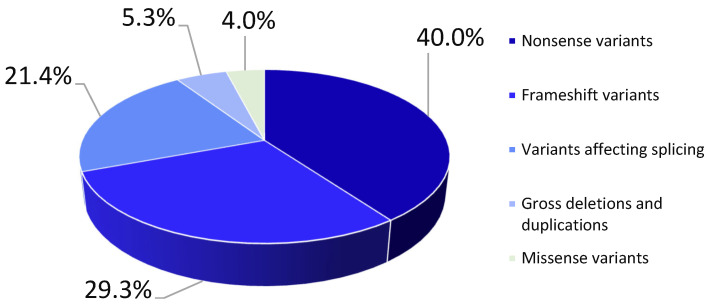
Types of mutations for pathogenic/likely pathogenic nucleotide sequence variants detected in the *LAMA2* gene in the examined probands.

In total, 75 pathogenic and likely pathogenic variants were detected: 40% (n = 30) were nonsense variants, 29.3% (n = 22) were frameshift variants, 21.4% (n = 16) affected splicing, 5.3% (n = 4) were gross deletions/duplications, and 4% (n = 3) were missense variants (Figure 1).

70 variants lead to the termination of protein synthesis, three variants result in amino acid substitution (c.163A>C, c.172T>C, c.2166A>T), and two variants affecting splicing lead to protein truncation (c.283+1G>A, c.3736-2A>T).

All previously unreported nucleotide sequence variants leading to the termination of protein synthesis were classified as pathogenic/likely pathogenic according to the American College of Medical Genetics and Genomics (ACMG) guidelines (https://www.acmg.net/docs/standards_guidelines_for_the_interpretation_of_sequence_variants.pdf, accessed on 5 December 2024). The missense variants c.163A>C, c.172T>C and c.2166A>T were also classified as likely pathogenic (PM2 (absent in controls (or at extremely low frequency if recessive), PM3 (for recessive disorders, detected in trans with a pathogenic variant), PP3 (multiple lines of computational evidence support a deleterious effect on the gene or gene product), PP4 (patient’s phenotype or family history is highly specific for a disease with a single genetic etiology). All three variants were absent in the Genome Aggregation Database (GnomAD v2.1.1, https://gnomad.broadinstitute.org/, accessed on 5 December 2024) and were identified in a trans position with a pathogenic variant.

Furthermore, biallelic combinations of different pathogenic or likely pathogenic variants (loss-of-function (LoF) variants) were identified in 51 patients for whom parental segregation analysis was not possible. According to the UK-ACGS Best Practice Guidelines for Variant Classification in Rare Disease 2020 (https://www.acgs.uk.com/quality/best-practice-guidelines/#VariantGuidelines, accessed on 5 December 2024), the variants detected in such cases could be regarded as a potential genetic cause of the disease, with a certainty exceeding 90% that each variant is pathogenic.

Five pathogenic/likely pathogenic variants in the *LAMA2* gene were the most common in the Russian Federation (Table 1). These variants were detected more than eight times. And they account for 41.1% (n = 74) of the total number of mutant alleles.

## 3. Discussion

In cases of MDC1A1, variants in the *LAMA2* gene typically lead to the termination of protein synthesis. Missense variants are rare and generally associated with a milder phenotype. The effect of a missense variant may be explained by its position in conservative sites and its impact on protein function (e.g., binding with α-dystroglycans, collagens, or influencing protein assembly). Similarly, in-frame deletions/duplications located in conservative sites can also result in a milder phenotype. Very rarely, a missense variant may result in a severe phenotype with merosin deficiency, in which case it is essential to confirm that the variant does not affect splicing.

In 83 of 90 cases, patients were diagnosed with the more severe form of *LAMA2*-associated muscular dystrophy. A total of 67 pathogenic/likely pathogenic variants were identified in this group: 29 nonsense variants, 20 frameshift variants, 15 splicing-affecting variants, and 3 gross deletions/duplications. No missense variants were detected in any of this group patients. In one case (patient 49), the nucleotide sequence variant c.5562+5G>C was identified (in a compound heterozygous state with a deletion of exon 1), a variant reported by I. Naom [11] in two patients with a milder clinical course of *LAMA2*-associated muscular dystrophy. In the first patient, this variant was identified in a compound heterozygous state with the c.5702del variant, in the second patient it was homozygous, and in both cases the variant c.5562+5G>C was detected in *cis* with c.9211+6T>C. In the first case, mRNA analysis of muscle tissue revealed no other transcripts, though the authors noted a decreased expression level of the normal transcript compared to control ones, suggesting nonsense-mediated decay. However, according to ClinVar data (https://www.ncbi.nlm.nih.gov/clinvar/, accessed on 5 December 2024) provided by the Greenwood Genetic Center Diagnostic Laboratories, this variant results in an 11-nucleotide insertion in intron 38. Functional studies conducted by Z. Tezak indicated that this variant, as well as the c.5562+5G>A variant, was observed in patients with a mild form of merosin-deficient MD in a heterozygous state (no second variants detected) and led to the formation of two transcripts—one with an insertion spanning 11 nucleotides and another with the deletion of exon 38 [12]. In the current study, a patient with this variant was consulted at the age of 38, with all medical history information provided by the proband’s mother. According to the mother, the first signs of the disease appeared at three weeks of life; the patient began holding her head up at six months, sat up at one year, but did not walk or crawl. In our case the second identified variant was the deletion of exon 1, leading to a frameshift. Unfortunately, functional analysis could not be carried out. It is possible that this patient predominantly has an isoform containing an 11-nucleotide insertion, which results in nonsense-mediated decay. It is also worth noting that this variant may cause either mild or severe forms of the disease, which corresponds with the data presented in the literature [5].

In patient 13, initial Sanger sequencing detected two variants in a compound heterozygous state: c.7074C>A (p.(Arg2578Ter)) and c.3412G>A (p.(Val1138Met)). The c.3412G>A variant was subsequently reclassified as benign, and MLPA analysis in this patient detected a deletion of exons 57 and 58, leading to a frameshift. This emphasizes that in case where a missense variant is identified in severe forms of merosin-deficient congenital muscular dystrophy, it is essential to ensure that no gross deletions/duplications or deep intronic variants are overlooked (own unpublished data). These variants can be detected using methods such as MLPA, target MPS panel including intronic region and whole-genome sequencing.

In 7 out of 90 cases, a milder clinical form of *LAMA2*-associated muscular dystrophy was diagnosed.

In the first case (Patient 84), pathogenic variants c.2049_2050del and c.3736-2A>T were identified in a compound heterozygous state [13,14,15]. The c.2049_2050del variant results in a frameshift and premature termination of protein synthesis. The c.3736-2A>T variant affects splicing, causing exon 26 skipping, which does not result in a frameshift but instead leads to the formation of a truncated protein. Exon 26 is located in domain IIIB, which is not a conservative region. The c.3736-2A>T variant has been previously reported as pathogenic in a homozygous state in a patient with a mild form of merosin-deficient muscular dystrophy [15].

In the second case (Patient 85), previously unreported variants ex40 del and c.172T>C were detected using whole-genome sequencing. However, no other pathogenic/likely pathogenic variants were found in other genes. The variant ex40 del leads to the premature termination of protein synthesis due to a frameshift, and is classified as pathogenic according to ACMG criteria (PVS1, PM2, PP4). The c.172T>C variant results in the replacement of cysteine with arginine at position 58 in domain VI, involved in merosin polymerization, which potentially explains the pathogenic effect of this variant. No additional pathogenic variants were found in the *LAMA2* gene, including deep intronic regions. The proband’s clinical data (including brain MRI) corresponded to *LAMA2*-associated muscular dystrophy, and the detected variants were located in trans position (variant c.172T>C inherited from mother and variant ex40 del inherited from father). Based on these data, the c.172T>C variant was classified as likely pathogenic (PM2, PP3, PM3, PP4) and may be associated with mild phenotype of the disease.

In the third case (Patient 86), exome sequencing revealed two compound heterozygous variants: a previously reported pathogenic variant c.1303C>T [16,17], which leads to premature translation termination, and a novel missense variant c.163A>C resulting in an asparagine-to-histidine substitution at position 55 in domain VI. No additional pathogenic/likely pathogenic variants or variants of uncertain clinical significance were found in the *LAMA2* gene (deep intronic regions were not analyzed). No other pathogenic/likely pathogenic variants were found in other genes as well. The clinical features of the disease (including brain MRI findings) were also consistent with *LAMA2*-associated muscular dystrophy, and the variants were identified in a trans position (variant c.163A>C inherited from mother and variant ex40 del inherited from father). Consequently, this variant was classified as likely pathogenic (PM2, PP3, PM3, PP4).

In the fourth case (Patient 87), whole-genome sequencing identified two variants in a compound heterozygous state: a LoF variant (c.7814del), not previously reported as pathogenic, leading to a frameshift and a missense variant (c.2166A>T), which was previously reported once [18], resulting in the substitution of glutamine with asparagine at position 722. According to ACMG criteria, the c.7814del variant was classified as pathogenic (PVS1, PM2, PP4). No other pathogenic or likely pathogenic variants were found in other genes. No additional pathogenic variants were found in the *LAMA2* gene, including deep intronic regions. The proband’s clinical features, including brain MRI findings, were consistent with *LAMA2*-associated muscular dystrophy, and the identified variants were located in a trans position (variant c.7814del inherited from mother and variant c.2166A>T inherited from father). Consequently, the c.2166A>T variant was classified as likely pathogenic (PM2, PP3, PM3, PP4) based on ACMG criteria.

In the fifth case (Patient 88), whole-genome sequencing revealed a variant (c.163A>C) identified in another patient in the current study, along with a novel insertion variant (c.8699_8700insGTAAATTCT), which causes termination of translation. No other pathogenic or likely pathogenic variants were found in other genes. No additional pathogenic variants were found in the *LAMA2* gene, including deep intronic regions. The variant c.8699_8700insGTAAATTCT was classified as pathogenic based on ACMG criteria (PVS1, PM2, PP4). The patient’s clinical findings, including their brain MRI, were consistent with *LAMA2*-associated muscular dystrophy. The variant was classified as likely pathogenic (PM2, PP3, PM3, PP4) based on ACMG guidelines.

In the sixth case (Patient 89), exome sequencing identified a previously reported pathogenic variant (c.4692_4695dup) and a novel variant (c.6721G>T), which leads to premature termination of translation. This novel variant was classified as pathogenic according to ACMG criteria (PVS1, PM2, PP4). Both identified variants were loss-of-function. A brain MRI revealed leukodystrophy, and immunohistochemical analysis of a muscle biopsy showed the absence of merosin. Despite these findings, the patient exhibited a mild form of merosin-deficient muscular dystrophy.

In the seventh case (Patient 90), exome sequencing revealed the c.8245-2A>G nucleotide sequence variant, previously reported once as pathogenic [19], in a homo-/hemizygous state. It was not possible to definitively determine the true zygosity of this variant. However, a consanguineous marriage of the proband’s parents allowed us to assume that this variant was in a homozygous state. The c.8245-2A>G variant has been reported in a single instance as pathogenic in a compound heterozygous state in a patient with a severe form of *LAMA2*-associated muscular dystrophy (MD) and in that case, IHC analysis showed residual expression of the laminin α2 chain and partial deficiency of merosin. The proband in this case exhibits a milder form of *LAMA2*-associated MD.

Thus, the missense variants identified in four cases can be regarded as mild, and it is possible that the presence of a single mild pathogenic variant in the patient’s genotype is sufficient to result in milder clinical symptoms. This observation is in accordance with the majority of global studies on merosin-deficient muscular dystrophy [3,20]. It is commonly known that loss-of-function (LoF) variants in homozygous or compound heterozygous states usually lead to a severe form of muscular dystrophy, whereas missense variants are predominantly associated with milder clinical findings. This may imply that pathogenic missense variants disrupt merosin protein assembly, leading to partial synthesis. Alternatively, missense variants may be localized in terminal domains, causing impaired merosin binding with α-dystroglycan and integrin, or affecting polymerization. However, exceptions to this rule also exist, in patients 89 and 90 with milder MD symptoms, two LoF variants were identified.

In case 89, IHC analysis revealed an absence of merosin protein despite the presence of two LoF variants, yet the patient had mild MD symptoms. This may be due to modifying factors, such as the expression of laminin α1 or laminin α5, which could influence the clinical presentation. Similar cases have been reported by A.A. Zambon [5] and P. Prandini [6]. In the case described by P. Prandini, a patient with a homozygous LoF variant exhibited a complete absence of laminin α2 on IHC, while overexpression of laminin α5 was observed. The expression of laminin β1 and laminin γ1 remained normal

Additionally, the presence of severe clinical symptoms in Patient 49, who carried a variant previously associated with milder forms, further supports the notion that despite the genotype–phenotype correlations proposed by various authors, exceptions to this rule exist [3,9]. However, other researchers did not observe genotype–phenotype correlations in their studies [5,21].

Five pathogenic/likely pathogenic variants in the *LAMA2* gene are the most common in the Russian Federation (Table 2). This list of recurring and frequent variants differs from those observed in other countries. In China, according to the data from Dandan Tan et al., the most frequent pathogenic variants among Han Chinese patients are c.7147C>T, del ex4 (*p* = 0.02), c.5156_5159del, c.2049_2050del, c.7921G>T, and c.4048C>T. Notably, the c.7536del variant was not observed at all in the Chinese cohort (*p* < 0.005). Additionally, a founder effect has been established for the del ex4 variant [20,22].

Among Spanish and Portuguese patients, the most common pathogenic variants were c.3085C>T (*p* < 0.05) and c.7750-1713_7899-2153del (del ex56) (*p* < 0.05) [23].

In Qatar, the most frequent pathogenic variant is c.6488del (*p* < 0.05), for which a founder effect has also been established [16].

The most frequent pathogenic variant in Russia was found to be the nucleotide sequence variant c.7536del, which was detected in 27 chromosomes out of 180. In four cases, this variant was detected in a homozygous state. In GnomAD v2.1.1 (https://gnomad.broadinstitute.org/, accessed on 5 December 2024) control cohort, the allelic frequency of this variant was 0.00001992 (5 alleles out of 250,958, with a frequency of 0.00001765 in the European non-Finnish population). According to the database GDB (Database of population frequencies of genetic variants among Russian patients. FMBA of Russia. App version 1.0.3, released on 5 December 2024. Base version 59.1, released on 3 October 2024. https://gdbpop.nir.cspfmba.ru/, accessed on 5 December 2024), the frequency of the c.7536del variant in Russia is 0.00043888 (106/241524) (*p* = 0.01). This variant had been reported Milovidova T. et al. as the most common pathogenic variant in the *LAMA2* gene in the Russian Federation, with a founder effect established for it [24].

The second most frequent variant was c.4692_4695dup, which caused a frameshift and premature termination of translation after 13 amino acid residues. The allelic frequency of the c.4692_4695dup variant, based on GnomAD v2.1.1 data, was 0.000003977 (1/251,424 in the European cohort). According to the database GDB, the frequency was 0.00008694788 (21/241,524) (*p* ≤ 0.05).

The c.8245-2A>G variant, which disrupts splicing, was not found in GnomAD v2.1.1. According to the database GDB, the frequency was 0.00007452676 (18/241,524). In the present study, this variant was exclusively observed among Kazan Tatars. The high frequency of this variant in this ethnic group may be attributed to the founder effect. In two cases, this variant was identified in a homozygous state: one case involved a consanguineous marriage, while no information regarding consanguinity was available in the second case.

The c.5116C>T variant, which leads to premature termination of translation, was observed with an allelic frequency of 0.00002483 (7/281,892) according to GnomAD v2.1.1 data (0.00003107 in the European non-Finnish population, 4/128,754). According to the database GDB the frequency was 0.0001407728 (34/241,524).

The c.2049_2050del variant, which results in a frameshift and premature termination of translation after 21 amino acid residues, was reported with an allelic frequency of 0.000116 (33/282,672) in the European cohort of GnomAD v2.1.1. According to the database GDB the frequency was 0.00006210563 (15/241,524).

**Figure 2:**
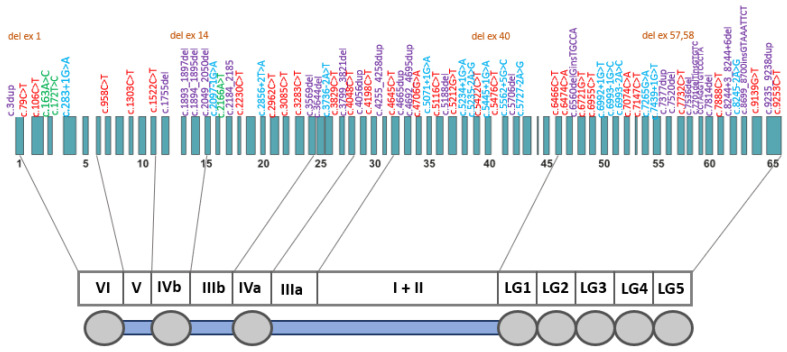
A schematic distribution of the pathogenic/likely pathogenic *LAMA2* gene (NM_000426) variants identified in this study. There was no strict correspondence between variants and exons. Variants are color-coded as follows: purple—frameshift variants; red—nonsense variants; blue—intronic variants affecting splicing; brown—gross deletions; green—missense variants.

Pathogenic/likely pathogenic variants were distributed across the entire *LAMA2* gene; most were located in exons 23–39 and exons 49–58 (Figure 2).

According to a recent study by Nicole J. Lake et al., the global birth prevalence of MDC1A1 is estimated to be 8.3 per million [25]. Population-specific prevalence estimations in GnomAD v2.1.1 range from 1.79 per million in East Asians to 10.1 per million in Europeans [25]. Additionally, Grazianu et al. reported an MDC1A1 prevalence of 0.563 per 100,000 in Italy [26], while Darin and Tulinius estimated the prevalence to be 2.5 per 100,000 in Western Sweden [27].

Based on the number of confirmed MDC1A1 cases in the Russian Federation (n = 83), the number of alleles with the most frequent pathogenic variant c.7536del (n = 27), and its frequency in the GDB database (0.00043888), the prevalence of MDC1A1 in the Russian Federation was estimated to be 1 in 137,000 (7 per million). The approximate calculated prevalence of *LAMA2*-associated muscular dystrophies (MDs) is 1 in 117,700. This estimation corresponds well to the range presented by Nicole J. Lake et al., which spans from 1.79 to 10.1 per million [25].

The geographic prevalence of MDC1A1 is highly variable: in Europe, it accounts for approximately 30% of all congenital muscular dystrophies (CMDs); in Japan, 6% [28]; in the UK, 37.4% [29]; in Italy, 24.1% [26]; and in Australia, 16% [30]. In Russia, according to the unpublished data from the DNA Diagnostics Laboratory of the FSBSI “RCMG”, MDC1A1 accounts for 30.8% of all CMD forms.

## 4. Materials and Methods

The study is retrospective; all patients were examined at the Research Centre for Medical Genetics (RCMG) and LLC “Genomed” between 2008 and 2024. The selection criteria for the study were as follows: the patient displayed clinical signs of muscular dystrophy, had two pathogenic/likely pathogenic variants in the *LAMA2* gene, and had provided signed informed consent (or consent was provided by a legal representative).

Molecular genetic analysis was carried out on genomic DNA samples extracted from peripheral blood lymphocytes according to standard manufacturer protocols. Pathogenic/likely pathogenic variants were identified by molecular genetic diagnostic methods, including mass parallel sequencing (targeted NGS panels), whole-exome sequencing, whole-genome sequencing (provided by LLC “Biotech Campus”, Moscow, Russia), and direct automated sequencing (sequencing of the *LAMA2* gene as the initial step in some cases, to confirm detected variants by NGS and to conduct family segregation analysis) with specific primers (see Supplementary Table S2). Additionally, a multiplex ligation-dependent probe amplification (MLPA) was carried out using the SALSA MLPA P391 *LAMA2* mix 1 and P392 *LAMA2* mix 2 (MRC-Holland, Amsterdam, The Netherlands). Mass parallel sequencing was carried out on next-generation sequencing platforms Ion S5™ (Thermo Scientific, Waltham, MA, USA), NextSeq 500 (Illumina, San Diego, CA, USA), DNBSEQ-T7 (MGI-Tech, Shenzhen, China), DNBSEQ-400 (MGI-Tech, China) and Genolab-M (GeneMind, Shenzhen, China). Sequencing data processing was carried out using the “NGS-Data” information system (https://ngs-data-ccu.epigenetic.ru, accessed on 5 October 2024). Variants were named according to the Human Genome Variation Society (HGVS) nomenclature (http://www.hgvs.org/mutnomen/, accessed on 5 December 2024), using nucleotide and amino acid numbering based on published coding reference DNA sequences (*LAMA2* NM_000426). All nucleotide sequence variants were compared with data published in the Human Genetic Mutation Database (HGMD Professional, 2024.3), the Leiden Muscular Dystrophy Database, and ClinVar. All variants were classified by pathogenicity based on the criteria of the American College of Medical Genetics and Genomics (ACMG).

Various patient groups, which included data obtained from the present study and data from studies conducted in other countries, were compared using two-sided Fisher’s exact test for categorical variables. All values with *p* < 0.05 were considered statistically significant for all tests used.

The frequency of *LAMA2*-associated dystrophy was determined based on the prevalence of the most common pathogenic variant, c.7536del, in Russia, according to GDB (Database of population frequencies of genetic variants among Russian patients. FMBA of Russia. App version 1.0.3, released on 5 December 2024. Base version 59.1, released on 3 October 2024. https://gdbpop.nir.cspfmba.ru/, accessed on 5 December 2024) and the number of alleles with this variant identified in patients with *LAMA2*-associated dystrophy.

## 5. Conclusions

We analyzed the clinical and molecular genetic data of Russian patients with *LAMA2*-associated muscular dystrophies (MDs). The spectrum of mutations in the *LAMA2* gene was established. Current research confirms that loss-of-function (LoF) variants are the most frequent cause of MDC1A1. A genotype–phenotype correlation was established; however, this correlation does not apply universally to all patients. Seven cases of milder form of *LAMA2*-associated muscular dystrophies (MDs) were confirmed in the current study. With that, the prevalence of LGMD associated with pathogenic/likely pathogenic variants in the *LAMA2* gene may be underestimated in Russia. The current study established that the most frequent pathogenic variants in the *LAMA2* gene in the Russian Federation are c.7536del and c.4692_4695dup. The most common pathogenic variant among Kazan Tatars is c.8245-2A>G. In Russia, the approximate calculated prevalence of *LAMA2*-associated muscular dystrophies (MDs) is 1 in 117,700. The present study may serve as a foundation for further investigations of the various aspects of *LAMA2*-associated muscular dystrophies all over the world.

## Figures and Tables

**Table 1:** The spectrum of detected pathogenic/likely pathogenic variants in the *LAMA2* gene (NM_000426.4) in the Russian Federation.

№	Exon/Intron	cDNA Position	Protein Change (NP_000417.3)	Number of Alleles/Prevalence	HGMD^®^ Professional (2024.3) ID/ACMG Criteria
1	54	c.7536del	p.(Asp2513IlefsTer34)	27/15.00%	CD2117468
2	32	c.4692_4695dup	p.(Arg1566CysfsTer13)	16/8.90%	CI054467
3	Intron 58	c.8245-2A>G	p.?	12/6.70%	CS1311884
4	36	c.5116C>T	p.(Arg1706Ter)	10/5.50%	CM981165
5	14	c.2049_2050del	p.(Arg683SerfsTer21)	9/5.00%	CD982727
6	55	c.7732C>T	p.(Arg2578Ter)	5/2.80%	CM032280
7	26	c.3829C>T	p.(Arg1277Ter)	4/2.20%	CM1816718
8	50	c.7147C>T	p.(Arg2383Ter)	4/2.20%	CM004723
9	65	c.9235_9238dup	p.(Thr3080AsnfsTer26)	4/2.20%	CI102066
10	49	c.6955C>T	p.(Arg2319Ter)	3/1.66%	CM981166
11	Intron 49	c.6992+1G>T	p.?	3/1.66%	PM2, PVS1, PP4
12	Intron 49	c.6993-2A>C	p.?	3/1.66%	CS085942
13	22	c.3085C>T	p.(Arg1029Ter)	3/1.66%	CM020725
14	46	c.6466C>T	p.(Arg2156Ter)	3/1.66%	CM142796
15	54	c.7520del	p.(Asn2507IlefsTer40)	3/1.66%	PM2, PVS1, PM3, PP4
16	2	c.163A>C	p.(Asn55His)	2/1.10%	PM2, PP3, PM3, PP4
17	Intron 2	c.283+1G>A	p.?	2/1.10%	CS102090
18	9	c.1303C>T	p.(Arg435Ter)	2/1.10%	CM102052
19	11	c.1522C>T	p.(Gln508Ter)	2/1.10%	CM2136520
20	14	c.1893_1897del	p.(Asp631GlufsTer8)	2/1.10%	CD021018
21	15	c.2184_2185del	p.(Gly729ValfsTer7)	2/1.10%	PM2, PVS1
22	14	del ex 14	p.?	2/1.10%	PM2, PVS1
23	32	c.4706G>A	p.(Trp1569Ter)	2/1.10%	PM2, PVS1
24	Intron 35	c.5071+1G>A	p.?	2/1.10%	CS151383
25	36	c.5212G>T	p.(Glu1738Ter)	2/1.10%	CM2136516
26	Intron 52	c.7439+1G>T	p.?	2/1.10%	PM2, PVS1
27	1	c.3dup	p.(Pro2AlafsTer48)	1/0.56%	PM2, PVS1
28	1	c.79C>T	p.(Gln27Ter)	1/0.56%	PM2, PVS1, PP4
29	1	c.106C>T	p.(Gln36Ter)	1/0.56%	PM2, PVS1
30	1	del ex 1	p.?	1/0.56%	CG1815550 CG1815547
31	2	c.172T>C	p.(Cys58Arg)	1/0.56%	CM1724080 PM2, PP3. PM3, PP4
32	7	c.958C>T	p.(Gln320Ter)	1/0.56%	PM2, PVS1, PP4
33	12	c.1755del	p.(Ser585ArgfsTer12)	1/0.56%	CD2124491
34	14	c.1894_1895del	p.(Leu632GlufsTer8)	1/0.56%	PM2, PVS1
35	Intron 14	c.2097-1G>A	p.?	1/0.56%	PM2, PVS1
36	15	c.2166A>T	p.(Glu722Asp)	1/0.56%	CM2214171 PM2, PP3, PM3, PP4
37	16	c.2230C>T	p.(Arg744Ter)	1/0.56%	CM130400 CS016102
38	Intron 20	c.2856+2T>A	p.?	1/0.56%	PM2, PVS1, PM3, PP4
39	21	c.2962C>T,	p.(Gln988Ter)	1/0.56%	CM981163
40	23	c.3283C>T	p.(Arg1095Ter)	1/0.56%	CM151370
41	25	c.3569del	p.(Ala1190ValfsTer9)	1/0.56%	PM2, PVS1, PM3, PP4
42	25	c.3644del	p.(Pro1215GlnfsTer9)	1/0.56%	PM2, PVS1
43	Intron 25	c.3736-2A>T	p.?	1/0.56%	CS2113419
44	26	c.3799_3821del	p.(Phe1267AspfsTer11)	1/0.56%	CG077478
45	27	c.4048C>T	p.(Arg1350Ter)	1/0.56%	CM102055
46	27	c.4056dup	p.(Arg1353GlnfsTer4)	1/0.56%	PM2, PVS1, PM3, PP4
47	29	C.4198C>T	p.(Arg1400Ter)	1/0.56%	CM1311877
48	29	c.4255_4258dup	p.(Cys1420SerfsTer5)	1/0.56%	CI243376
49	32	c.4645C>T	p.(Arg1549Ter)	1/0.56%	CM001209
50	32	c.4665dup	p. (Lys1556GlufsTer3)	1/0.56%	PM2, PVS1
51	36	c.5188del	p.(Arg1730GlyfsTer4)	1/0.56%	PM2, PVS1, PM3
52	Intron 36	c.5234+1G>A	p.?	1/0.56%	CS085941
53	Intron 36	c.5235-2A>G	p.?	1/0.56%	PM2, PVS1, PM3, PP4
54	37	c.5422C>T	p.(Gln1808Ter)	1/0.56%	PM2, PVS1, PM3, PP4
55	Intron 37	c.5445+1G>A	p.?	1/0.56%	CS206309
56	38	c.5476C>T	p.(Arg1826Ter)	1/0.56%	CM983961
57	Intron 38	c.5562+5G>C	p.?	1/0.56%	CS003701
58	Intron 38	c.5727-2A>G	p.?	1/0.56%	PM2, PVS1
59	39	c.5706del	p.(Ser1903HisfsTer61)	1/0.56%	PM2, PVS1
60	40	del ex 40	p.?	1/0.56%	PM2, PVS1, PP4
61	46	c.6474C>A	p.(Tyr2158Ter)	1/0.56%	PM2, PVS1, PP4
62	46	c.6560delinsTGCCA	p.(Gly2187ValfsTer8)	1/0.56%	PM2, PVS1, PP4
63	48	c.6721G>T	p.(Gly2241Ter)	1/0.56%	PM2, PVS1, PP4
64	Intron 49	c.6993-1G>C	p.?	1/0.56%	PM2, PVS1, PP4
65	50	c.7074C>A	p.(Tyr2358Ter)	1/0.56%	CM981167
66	51	c.7265G>A	p.(Trp2422Ter)	1/0.56%	PM2, PVS1
67	52	c.7377dup	p.(Leu2460SerfsTer2)	1/0.56%	CI020898
68	55	c.7701delTinsGTGTCCCTAGGTGTCCCTA	p.(Ser2567delinsArgCysProTer)	1/0.56%	PM2, PVS1, PM3, PP4
69	56	c.7814del	p.(Thr2605LysfsTer2)	1/0.56%	PM2, PVS1, PP4
70	56	c.7888C>T	p.(Arg2630Ter)	1/0.56%	CM1618984
71	57,58	del ex 57,58	p.?	1/0.56%	PM2, PVS1, PP4
72	58	c.8244+3_8244+6del	p.?	1/0.56%	CD151394
73	61	c.8699_8700insGTAAATTCT	p.(Pro2901Ter)	1/0.56%	PM2, PVS1, PP4
74	64	c.9139G>T	p.(Glu3047Ter)	1/0.56%	CM2320779
75	65	c.9253C>T	p.(Arg3085Ter)	1/0.56%	CM020949
			Total	180/100%	

**Table 2:** Most common pathogenic variants in the *LAMA2* gene (NM_000426.4), according to the data obtained during the current study.

№	cDNA Position	Protein Change (NP_000417.3)	Number of Alleles/Prevalence	Allele Frequency GnomAD (V2.1.1)	Allele Freguency GDB (V59.1)
1	c.7536del	p.(Asp2513IlefsTer34)	27/15.00%	0.000019	0.00043888
2	c.4692_4695dup	p.(Arg1566CysfsTer13)	16/8.90%	0.000004	0.000086948
3	c.8245-2A>G	Splice	12/6.70%	n/d	0.000074527
4	c.5116C>T	p.(Arg1706Ter)	10/5.50%	0.000024	0.000140773
5	c.2049_2050del	p.(Arg683SerfsTer21)	9/5.00%	0.000116	0.000062106

## Data Availability

Data are contained within the article.

## Supplementary Materials

The supporting information can be downloaded at: https://www.mdpi.com/article/10.3390/ijms26031257/s1.
